# Mediation of the association between physical activity and metabolic dysfunction-associated fatty liver disease in children and adolescents by inflammatory markers: an analysis of 2017‒2020 NHANES data

**DOI:** 10.1016/j.clinsp.2026.101056

**Published:** 2026-07-16

**Authors:** Chunyang Zhang, Xiaonan Li

**Affiliations:** a School of Pediatrics, Nanjing Medical University, Nanjing, Jiangsu, China; b Department of Pediatric Surgery, The Affiliated Lianyungang Hospital of Xuzhou Medical University, Lianyungang, Jiangsu, China; c Department of Pediatric Surgery, The First Affiliated Hospital of Kangda College of Nanjing Medical University, Lianyungang, Jiangsu, China

**Keywords:** Inflammatory markers, Physical activity (PA), Metabolic dysfunction-associated fatty liver disease (MAFLD), Children and adolescents, Mediation analysis

## Abstract

**Background:**

Metabolic dysfunction-Associated Fatty Liver Disease (MAFLD) is a globally prevalent condition that is closely related to Physical Activity (PA). Inflammatory mediators play a pivotal role in the pathophysiology of both MAFLD and PA. This study aimed to elucidate the association between PA and MAFLD in children and adolescents and to assess the mediating role of inflammatory markers in this relationship based on 2017‒2020 National Health and Nutrition Examination Survey (NHANES) data.

**Methods:**

In this cross-sectional study, participants aged < 18 were included, with relevant data derived from the NHANES 2017‒2020 cycles. Weighted univariate and multivariate logistic regression models, together with subgroup analyses, were employed to examine the association between PA and MAFLD prevalence. Mediation analysis was conducted to evaluate the potential intermediary role of inflammatory markers in this relationship.

**Results:**

979 children and adolescents were included, with an overall MAFLD prevalence of 24.96%. In multivariable-adjusted models, participants who met PA recommendations exhibited a significantly lower prevalence of MAFLD compared with those who did not (OR = 0.45; 95% CI 0.23‒0.87). Mediation analyses revealed that monocyte count, segmented neutrophil count, and lymphocyte count accounted for 7.72%, 8.79%, and 0.52% of the association between PA and MAFLD prevalence, respectively.

**Conclusion:**

Among children and adolescents younger than 18, higher levels of PA were significantly and inversely associated with the presence of MAFLD. This association appears to be partially mediated by inflammatory pathways, suggesting that the protective effect of PA against MAFLD may, at least in part, be attributable to its capacity to modulate systemic inflammation.

## Highlights

• It examined the mediating role of inflammatory markers in the association of PA with MAFLD risk in children and adolescents.
• This study clarified the mediating role of inflammatory markers in the relation of PA to MAFLD risk.
• This study may inform the development of evidence-based health promotion policies and tailored public health strategies.

## Introduction

Childhood Metabolic dysfunction-Associated Fatty Liver Disease (MAFLD) is a pressing public health challenge, and its incidence has risen sharply in recent years. This trend parallels the rising prevalence of pediatric obesity, a condition that not only poses immediate health risks but also predisposes affected individuals to long-term metabolic complications, including Type 2 Diabetes Mellitus (T2DM) and cardiovascular disease. Current epidemiological evidence indicates a substantial burden of MAFLD among children, underscoring the urgent need for effective screening strategies and targeted interventions to mitigate its impact on both pediatric populations and their families.^1^^,^^2^

Physical Activity (PA) is pivotal in maintaining both physical and psychological health.^3^ Increasing evidence suggests a strong association between insufficient PA and the development of MAFLD. Sedentary behavior, in particular, has been implicated in ectopic fat deposition, an established independent risk factor for MAFLD.^4^ For example, a study conducted in South Korea demonstrated that sedentary lifestyles are associated with hepatic injury and confer approximately a 20% higher risk of MAFLD.^5^ It has been consistently observed that individuals with MAFLD engage in significantly lower levels of PA than their non-MAFLD counterparts. PA has been shown to reduce hepatic fat accumulation and improve insulin sensitivity, among other metabolic benefits. Previous studies have demonstrated an inverse association between PA and MAFLD risk.^6^ However, most existing research has focused on adult populations or simple associations, with limited investigation into the underlying biological mechanisms, particularly the role of inflammation.

Inflammation is widely recognized as a key driver of MAFLD progression and hepatic fibrogenesis. This is evidenced by the strong association between Nonalcoholic Steatohepatitis (NASH) and advanced fibrosis, including stage 4 disease. Inflammatory pathways are involved throughout the entire spectrum of Nonalcoholic Fatty Liver Disease (NAFLD), particularly in advanced stages such as cirrhosis and progression to Hepatocellular Carcinoma (HCC).^7^, ^8^, ^9^, ^10^, ^11^, ^12^, ^13^, ^14^, ^15^, ^16^ Hepatic inflammation reflects disease progression, and numerous mechanistic studies in animal models have demonstrated that immune cell activity promotes liver fibrosis and the transition to NASH.^10^ Recent research has highlighted the complex interplay between inflammation and MAFLD. Neutrophils, as key components of the innate immune system, play a central role in these processes. In the early stages of MAFLD, suppression of neutrophil activity can reduce serum Alanine Aminotransferase (ALT) levels and attenuate hepatic inflammation as well as proinflammatory gene expression. Huang et al. further elucidated the role of neutrophils in MAFLD pathogenesis.^12^ Increasing evidence indicates that neutrophil infiltration into hepatic tissue exacerbates hepatocellular injury through the excessive release of inflammatory mediators, thereby amplifying hepatic inflammation and fibrogenesis.^13^^,^^14^

To date, studies investigating the potential mediating role of inflammation-related biomarkers in the association between PA and MAFLD prevalence, particularly in pediatric populations, remain limited. Inflammatory markers may serve as important mediators in this relationship and could provide a mechanistic explanation for the protective effects of PA against MAFLD. Elucidating this pathway may enhance understanding of MAFLD pathogenesis and facilitate the development of more precise and effective prevention strategies for at-risk children.

## Materials and methods

### Study design and population

The present study utilized data from the National Health and Nutrition Examination Survey (NHANES), an ongoing, nationally representative program assessing the health and nutrition of the civilian, noninstitutionalized Americans (https://wwwn.cdc.gov/nchs/nhanes/).

NHANES employs a complex, stratified, multistage probability sampling design to ensure that the study population accurately represents the demographic characteristics of the U.S. population.^14^ This analysis was based on publicly available, de-identified NHANES data. The original NHANES protocols were approved by the relevant ethics review boards, and written informed consent was obtained from all participants or their legal guardians (for children and adolescents).^15^ Therefore, no additional ethical approval was required for this secondary analysis.

**Fig. 1:**
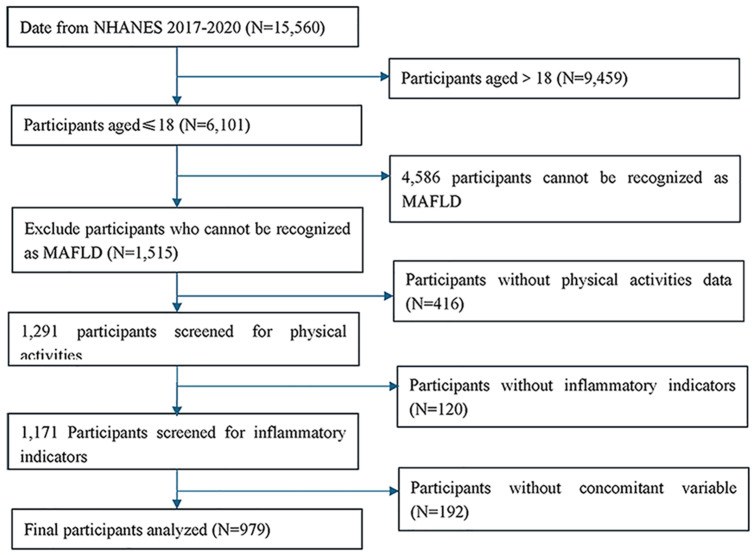
Flowchart of study population inclusion.

15,560 individuals were identified from the 2017‒2020 NHANES cycles (Fig. 1). To ensure analytical rigor and validity, the following exclusion criteria were applied: 1) Age > 18; 2) Absence of a confirmed MAFLD diagnosis; 3), Missing PA questionnaire data; 4) Lack of inflammatory marker data; and 5) Missing covariate information, including sex, age, and race.

### Evaluation of PA

PA levels were assessed through the Physical Activity Questionnaire (PAQ). Participants were asked: “On how many days during the past week did you engage in exercise, sports, or PA for at least 60-minutes?” Response options included 0-days, 1‒3 days, 4‒6 days, or every day. Participants reporting daily PA were classified as meeting PA guidelines, whereas all others were categorized as not meeting the guidelines.^10^

### Evaluation of MAFLD

MAFLD status was determined using transient elastography to assess Hepatic Steatosis (HS). In children, MAFLD was defined as the presence of HS with one or more of the following conditions: excess adiposity (overweight/obesity or abdominal obesity), prediabetes, T2DM, or metabolic abnormalities. According to World Health Organization (WHO) criteria, a Body Mass Index (BMI) *z*-score ≥ 1 Standard Deviation (SD) indicates overweight or obesity in children aged 5‒10. Abdominal obesity was defined as a waist circumference exceeding the age- and sex-specific 90th percentile. Prediabetes was defined as Fasting Plasma Glucose (FPG) of 5.6‒6.9 mmoL/L or glycated Hemoglobin (HbA1c) of 5.7%‒6.4%, whereas T2DM was defined as FPG ≥7.0 mmoL/L or HbA1c ≥6.5%. For children aged 10‒15, metabolic abnormalities were defined by the presence of at least two of the following: 1) Systolic blood pressure >130 mmHg or diastolic blood pressure > 85 mmHg; 2) Triglycerides (TG) > 150 mg/dL; 3) High-density Lipoprotein Cholesterol (HDL-C) < 40 mg/dL; and 4) TG/HDL-C ratio > 2.25. For participants aged ≥ 15, adult diagnostic criteria for MAFLD were applied.^17^

### Evaluation of inflammatory indicators

Hematological parameters were obtained from the NHANES Complete Blood Count (CBC) dataset using the DxH 900 analyzer (Beckman Coulter, Brea, CA, USA), including measurements of monocytes, segmented neutrophils, lymphocytes, and platelets. White blood cell differentials were determined using the Coulter Volume, Conductivity, and Scatter (VCS) system. The analyzer performs automated dilution and mixing during sample processing. Hemoglobin concentration was measured using single-beam photometry.

### Covariates

Sociodemographic and lifestyle variables were included as covariates based on prior literature, including age, sex, race/ethnicity, smoking status, and the Poverty-to-Income Ratio (PIR). Household smoking exposure was categorized as no smokers, one smoker, or two or more smokers in the household. Additional covariates included serum HDL-C concentration (mmoL/L), total fat intake (g/day), dietary fiber intake (g/day), total sugar intake (g/day), and other relevant dietary variables.

### Statistical analysis

Given the complex, multistage sampling design of NHANES, all analyses incorporated sample weights, stratification, and clustering to ensure nationally representative estimates.

Continuous variables with a normal distribution were expressed as means ± SD, whereas non-normally distributed variables were presented as medians with Interquartile Ranges (IQRs) [M (Q1, Q3)]. Between-group comparisons were conducted using the Student’s *t*-test or the Mann-Whitney *U*-test, as appropriate. Categorical variables were expressed as counts and percentages [n (%)], and comparisons were performed using the Chi-Square (χ^2^) test or Fisher’s exact test. Multicollinearity among covariates was assessed using the Variance Inflation Factor (VIF), with all included variables showing VIF values < 5, indicating no significant multicollinearity. The association between PA and MAFLD was examined using univariate and multivariate logistic regression models. Model 1 was unadjusted; Model 2 was adjusted for age, sex, and race/ethnicity; and Model 3 was further adjusted for PIR, serum cotinine level, household smoking status, serum HDL-C, total fat intake, dietary fiber intake, total sugar intake, and other relevant variables.

Potential effect modification and population heterogeneity were explored through subgroup analyses. Mediation analysis was conducted to evaluate whether inflammatory markers (monocyte count, segmented neutrophil count, lymphocyte count, and platelet count) mediated the association between PA and MAFLD status. The analysis followed a causal mediation framework and was performed using the mediation package in R to estimate total, direct, and indirect effects of PA on MAFLD through each inflammatory marker. Statistical significance and 95% Confidence Intervals were derived using robust standard errors based on 1000 simulations.

Finally, sensitivity analyses were conducted by additionally adjusting for ALT, Aspartate Aminotransferase (AST), creatinine, adolescent hypertension, and diabetes to assess the robustness of the findings.

The present statistical analyses were completed using R 4.3.2. Two-sided p < 0.05 denotes statistical significance. All analyses accounted for the complex NHANES survey design, including sample weights, stratification, and clustering, to produce nationally representative estimates and valid standard errors.

## Results

### Baseline participant characteristics

979 participants were included in the analysis. The overall prevalence of MAFLD was 24.96%. The median age was 15 [IQR: 13‒16]. Among the participants, 474 (49.69%) were male, and 505 (50.31%) were female. In terms of ethnicity, 414 participants (34.62%) were Mexican American, while 565 (65.38%) belonged to other racial/ethnic groups. Regarding household smoking status, 664 participants (71.43%) lived in non-smoking households, 189 (16.59%) in households with one smoker, and 126 (11.98%) in households with two or more smokers. The median total energy intake was 1853 kcal (IQR: 1414‒2651), total protein intake was 63.76 g (IQR: 44.63‒94.04), carbohydrate intake was 233.85 g (IQR: 172.65‒334.12), total sugar intake was 96.36 g (IQR: 62.66‒145.98), and dietary fiber intake was 12.8 g (IQR: 8.50‒18.10). Participants were stratified into MAFLD and non-MAFLD groups. Significant differences between the groups were observed in race/ethnicity, serum cotinine levels, total energy intake, and carbohydrate intake (all p < 0.05). Notably, PA levels differed significantly between the two groups (p = 0.009) (Table 1).

**Table 1:** Baseline participant characteristics by childhood MAFLD status.

**Characteristic**	**Overall**	**Normal**	**MAFLD**	**p-value**
Age	15.00 (13.00, 16.00)	15.00 (13.00, 16.00)	15.00 (13.00, 16.00)	0.700
Gender				0.200
Female	474 (49.69%)	359 (51.19%)	115 (45.20%)	
Male	505 (50.31%)	369 (48.81%)	136 (54.80%)	
Race				0.004
Mexican American	414 (34.62%)	293 (31.32%)	121 (44.54%)	
Other Hispanic	565 (65.38%)	435 (68.68%)	130 (55.46%)	
PIR	2.52 (1.22, 4.27)	2.89 (1.36, 4.64)	1.78 (0.87, 3.32)	<0.001
Smoking				0.600
0 No one in the household is a smoker	664 (71.43%)	502 (72.26%)	162 (68.94%)	
1 household member is a smoker	189 (16.59%)	136 (16.41%)	53 (17.13%)	
2 or more household members are smokers	126 (11.98%)	90 (11.34%)	36 (13.93%)	
Cotinine, Serum (ng/mL)				0.007
TSE	443 (38.74%)	309 (35.02%)	134 (49.95)	
No TSE	536 (61.26%)	419 (64.98%)	117 (50.05%)	
Energy (kcal)	1853.00 (1414.00, 2651.00)	1915.00 (1428.00, 2753.00)	1744.00 (1313.00, 2328.00)	0.005
Protein (gm)	63.76 (44.63, 93.04)	63.81 (45.56, 94.90)	63.54 (40.88, 84.07)	0.085
Carbohydrate (gm)	233.85 (172.65, 334.12)	239.85 (175.61, 337.57)	209.37 (152.30, 303.50)	0.013
Total sugars (gm)	93.36 (62.66, 145.98)	98.50 (63.03, 145.98)	87.91 (61.10, 146.04)	0.300
Dietary fiber (gm)	12.80 (8.50, 18.10)	13.10 (9.10, 18.20)	11.80 (7.50, 16.90)	0.062
PA				0.009
Not meet	832 (85.08%)	607 (83.16%)	225 (90.85%)	
Meet	147 (14.92%)	121 (16.84%)	26 (9.15%)	

Note: MAFLD, Metabolic dysfunction-Associated Fatty Liver Disease; Normal, Non-MAFLD; Overall, All participants.

### LR model

The association between PA and MAFLD risk was examined using three logistic regression models. Using participants who did not meet the recommended PA levels as the reference group, those who met the PA recommendations exhibited a significantly lower prevalence of MAFLD (Model 1: OR = 0.50, 95% CI: 0.30‒0.83; Model 2: OR = 0.50, 95% CI: 0.29‒0.88; Model 3: OR = 0.45, 95% CI: 0.23‒0.87) (Table 2).

**Table 2:** Association between PA and MAFLD.

**Participants**	**Model 1**		**Model 2**		**Model 3**	
	**OR (95% CI)**	**p-value**	**OR (95% CI)**	**p-value**	**OR (95% CI)**	**p-value**
PA						
Group						
Not meet	Ref		Ref		Ref	
Meet	0.50 (0.30, 0.83)	0.01	0.50 (0.29, 0.88)	0.019	0.45 (0.23, 0.87)	0.021

Note: Model 1 encompassed PA as the sole predictor to assess its association with MAFLD in children. Model 2 adjusted for PA, sex, age, and race. Model 3 encompassed all covariates like PA, sex, age, race, serum cotinine levels, and dietary factors.

### Subgroup analyses

Subgroup analyses by sex, race, smoking status, and serum cotinine were carried out. Using the group that did not meet PA recommendations as the reference, a statistically significant inverse association between PA and MAFLD risk was observed in specific subgroups, particularly among males and Hispanic participants (Table 3). However, interaction analyses indicated no statistically significant interactions between PA and these stratification variables with respect to MAFLD risk (all p for interaction >0.05).

**Table 3:** Association between PA and MAFLD stratified by subgroups.

**Subgroup**	**n**	**OR (95% CI)**		**p-value**	**p for interaction**
		**Not meet**	**Meet**		
Gender					0.326
Female	115/474	Ref	0.63 (0.16, 2.52)	0.5	
Male	136/505	Ref	0.39 (0.25, 0.69)	0.001	
Race					0.186
Mexican American	121/414	Ref	0.32 (0.15, 0.67)	0.005	
Other Hispanic	130/565	Ref	0.57 (0.22, 1.48)	0.2	
Smoking					0.386
0 No one in the household is a smoker	162/664	Ref	0.54 (0.22, 1.33)	0.2	
1 household member is a smoker	53/189	Ref	0.41 (0.10, 1.75)	0.2	
2 or more household members are smokers	36/126	Ref	0.18 (0.03, 1.19)	0.065	
Cotinine, Serum (ng/mL)					0.266
TSE	134/443	Ref	0.39 (0.20, 0.78)	0.011	
No TSE	117/536	Ref	0.63 (0.20, 1.96)	0.4	

Note: Subgroup analyses were based on Model 3, adjusting for all covariates, including PA, sex, age, race, serum cotinine levels, and dietary factors.

### Mediation analysis

**Fig. 2:**
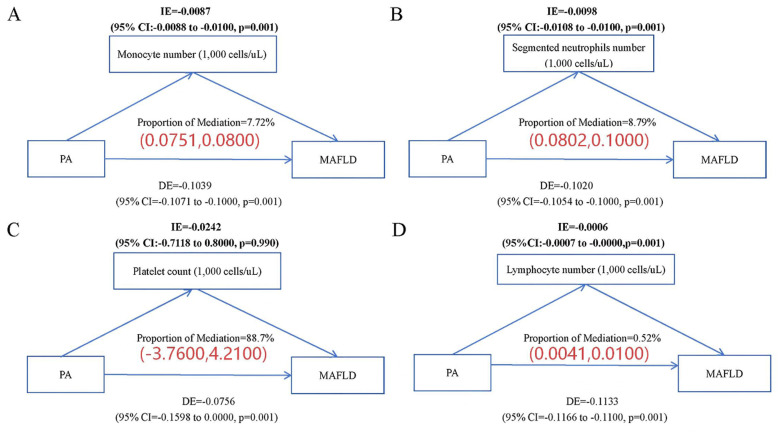
Path diagram illustrating the mediating effect of inflammatory markers on the association between PA and MAFLD. (A‒D) The icons respectively represent the mediating roles of monocytes, segmented neutrophils, lymphocytes, and platelets. IE, Indirect Effect; DE, Direct Effect; MAFLD, Metabolic dysfunction-Associated Fatty Liver Disease.

The potential mediating role of inflammation-related markers in the association between PA and MAFLD risk was evaluated using mediation models. Monocyte count, segmented neutrophil count, and lymphocyte count were found to partially mediate this association, with mediation proportions of 7.72%, 8.79%, and 0.52%, respectively (Figs. 2A, B, and D). No significant mediating effect was observed for platelet count (Fig. 2C).

### Sensitivity analysis

To assess the robustness of the findings, sensitivity analyses were conducted with additional adjustment for ALT, AST, creatinine, adolescent hypertension, and diabetes. The results remained consistent, indicating the robustness of the main findings (Supplementary Table 1).

## Discussion

MAFLD, a liver-impacting symptom of multisystem metabolic dysfunction, currently compromises the health of one-third of people worldwide. The present study systematically examined the interrelationships among PA, MAFLD, and inflammatory mediators. These findings demonstrated a significant inverse association between PA and MAFLD prevalence. Compared with children and adolescents with insufficient PA, those who met recommended PA levels exhibited a markedly lower prevalence of MAFLD. Inflammatory mediators appeared to partially mediate this association, with monocytes, segmented neutrophils, and lymphocytes accounting for 7.72%, 8.79%, and 0.52% of the mediation effect, respectively.

A substantial body of evidence in adult populations has shown that sedentary behavior and insufficient PA are closely associated with the onset and progression of MAFLD. The present study extends these findings to children and adolescents, demonstrating that higher levels of PA are significantly associated with a lower prevalence of MAFLD. These results further support the potential role of lifestyle interventions in the early prevention of metabolic liver disease. Previous studies have suggested that PA may confer metabolic benefits by improving insulin sensitivity, reducing hepatic fat accumulation, and alleviating chronic low-grade inflammation. The well-established association between childhood obesity, hepatic fat deposition, and insulin resistance also indirectly supports these mechanisms. However, it should be noted that the present study is cross-sectional in nature; thus, the proposed mechanisms are largely inferred from prior research and require further validation. Importantly, childhood and adolescence represent critical periods for the establishment of metabolic patterns and lifestyle behaviors, yet research on MAFLD in this population remains limited. The present findings suggest that achieving recommended levels of PA may help reduce MAFLD risk, underscoring the public health importance of promoting PA early in life.^18^, ^19^, ^20^

A key finding of this study is that inflammatory cell counts partially mediated the association between PA and MAFLD in children and adolescents, supporting a potential biological pathway linking lifestyle behaviors to metabolic liver disease. Specifically, PA may reduce systemic low-grade inflammation through multiple mechanisms, including decreasing visceral adiposity, improving immune regulation, and suppressing the production of proinflammatory cytokines. In turn, chronic inflammation is known to contribute to MAFLD development and progression by promoting insulin resistance, altering hepatic lipid metabolism, and activating liver-resident immune cells. In the present analysis, monocytes and neutrophils contributed more substantially to the mediation effect than lymphocytes, suggesting that innate immune responses may play a more prominent role in this pathway. This observation is consistent with existing evidence indicating that innate immune activation is an early driver of metabolic inflammation. However, the relatively modest mediation proportions observed indicate that inflammation explains only a limited part of the association. Other mechanisms, such as insulin resistance, adipokine dysregulation, and ectopic fat deposition, are likely to play more substantial roles. Therefore, the relationship between PA and MAFLD is likely mediated by multiple interconnected biological pathways rather than a single inflammatory mechanism.^21^, ^22^, ^23^

Subgroup analyses were further conducted to explore potential heterogeneity in the association between PA and MAFLD. The inverse association between PA and MAFLD risk was generally consistent across strata defined by sex, race/ethnicity, smoking exposure, and serum cotinine levels. However, no statistically significant interactions were observed (all p for interaction >0.05), suggesting the absence of clear effect modification. Although stronger associations were observed in certain subgroups (e.g., males or Mexican American participants), these differences may be attributable to variations in sample size or statistical fluctuation rather than true biological heterogeneity. Therefore, these findings should be interpreted with caution, and overinterpretation of subgroup-specific effects should be avoided.^24^, ^25^, ^26^

There are some limitations. First, fatty liver was diagnosed using transient elastography rather than histological confirmation, which remains the gold standard. Second, although extensive adjustments were made for potential confounders, residual confounding cannot be entirely ruled out. Third, PA was evaluated באמצעות self-reported questionnaires, which may be subject to recall bias and misclassification. Finally, the study population consisted exclusively of U.S. participants, which may limit the generalizability of the findings to other racial, ethnic, or geographic populations. Future studies employing longitudinal designs and incorporating more specific inflammatory biomarkers, such as cytokines and immune cell phenotypes, are warranted to clarify temporal relationships and underlying mechanisms. In addition, the application of more advanced analytical approaches, including multiple or competing mediation models, may further elucidate the relative contributions of distinct biological pathways.

## Conclusion

Inflammatory mediators may serve as potential predictive biomarkers for MAFLD in children and adolescents. Regular engagement in PA appears to mitigate systemic inflammation, thereby reducing the risk of MAFLD. These findings highlight the importance of early identification of high-risk populations and support the promotion of age-appropriate PA through targeted and feasible interventions. Such measures could be incorporated into public health strategies for reducing the burden of MAFLD in the pediatric population.

## Abbreviations

ALT, Alanine Aminotransferase; AST, Aspartate Aminotransferase; BMI, Body Mass Index; FPG, Fasting Plasma Glucose; HbA1c, Hemoglobin; HCC, Hepatocellular Carcinoma; HDL-C, High-Density Lipoprotein Cholesterol; HS, Hepatic Steatosis; IQR, Interquartile Range; LR, Logistic Regression; MAFLD, Metabolic Dysfunction-Associated Fatty Liver Disease; NAFLD, Nonalcoholic Fatty Liver Disease; NASH, Nonalcoholic Steatohepatitis; NHANES, National Health and Nutrition Examination Survey; PA, Physical Activity; PIR, Poverty-to-Income Ratio; T2DM, Type 2 Diabetes Mellitus; TG, Triglycerides; VCS, Volume, Conductivity, and Scatter; WHO, World Health Organization.

## Authors’ contributions

**Chunyang Zhang:** Conceptualization, Methodology, Formal Analysis, Investigation, Resources, Writing – Original Draft, Writing – Review & Editing, Supervision, Validation, Project Administration. **Xiaonan Li:** Formal Analysis, Investigation, Writing – Review & Editing, Validation. All authors read and approved the final manuscript.

## Ethical statement

All study procedures were conducted as per the Declaration of Helsinki. This study utilized publicly available NHANES data and was approved by the NCHS Research Ethics Review Committee. Every participant gave informed consent in writing. Ethical review was exempted for the present analysis due to the use of de-identified public data.

## Funding

This research did not receive any specific grant from funding agencies in the public, commercial, or not-for-profit sectors.

## Data availability

The datasets analyzed are publicly available from the NCHS at: https://www.cdc.gov/nchs/nhanes/about/index.html.

## Conflicts of interest

The authors declare no conflicts of interest.

## Appendix Supplementary materials

Image, application 1
