# Supplementary material for: Mediation of the association between physical activity and metabolic dysfunction-associated fatty liver disease in children and adolescents by inflammatory markers: an analysis of 2017‒2020 NHANES data
Source: Clinics (Sao Paulo). 2026 Jul 16;81:101056. doi: 10.1016/j.clinsp.2026.101056 (PMC13401007; doi:10.1016/j.clinsp.2026.101056)
Supplement: Supplementary file 1 [file mmc1.docx]

**CLINICS-D-26-00038**

**Supplementary Materials**

**Supplementary Table 1** Association between PA and MAFLD.

| **Participants** | **Model 1** | | **Model 2** | | **Model 3** | | **Model 4** | | **Model 5** | |
| --- | --- | --- | --- | --- | --- | --- | --- | --- | --- | --- |
|  | **OR (95%CI)** | **p-value** | **OR (95%CI)** | **p-value** | **OR (95%CI)** | **p-value** | **OR (95%CI)** | **p-value** | **OR (95%CI)** | **p-value** |
| PA |  |  |  |  |  |  |  |  |  |  |
| Group |  |  |  |  |  |  |  |  |  |  |
| Not meet | Ref |  | Ref |  | Ref |  | Ref |  | Ref |  |
| Meet | 0.50 (0.30, 0.83) | 0.010 | 0.50 (0.29, 0.88) | 0.019 | 0.45 (0.23, 0.87) | 0.021 | 0.48 (0.26, 0.91) | 0.028 | 0.48 (0.24, 0.94) | 0.036 |

Note: Model 4: adjusted for all covariates, including sex, age, race, serum cotinine levels, dietary factors, ALT, AST, and creatinine. Model 5: adjusted for all covariates, including sex, age, race, serum cotinine levels, dietary factors, ALT, AST, creatinine, hypertension, and juvenile diabetes.
